# From North American hegemony to global competition for scientific leadership? Insights from the Nobel population

**DOI:** 10.1371/journal.pone.0213916

**Published:** 2019-04-03

**Authors:** Thomas Heinze, Arlette Jappe, David Pithan

**Affiliations:** 1 Interdisciplinary Center of Science and Technology Studies (IZWT), University of Wuppertal, Wuppertal, Germany; 2 Institute of Sociology, University of Wuppertal, Wuppertal, Germany; Lancaster University, UNITED KINGDOM

## Abstract

Based on the entire population of Nobel laureates in science from 1901 to 2017, we show that North America’s rise as global power in science started in the 1920s. Following a transition period (1940s to 1960s), its scientific hegemony was consolidated in the 1970s. Yet since the 2000s, North America’s global leadership in science has come under pressure. In that time, its share of laureates across disciplines dropped, although it has retained its attractiveness as a destination for future laureates from Europe and the Asia-Pacific region. In addition, we find that North America has become apparently less effective since 2010 in transferring capacities for conducting ground-breaking research from one generation of scientists to another. Furthermore, both Europe and the Asia-Pacific region have similarly high shares of newcomer organizations with regard to where prize-winning work is conducted, indicating that these two regions are very active in the inter-organizational competition for scientific talent. Despite this competition, however, we find no support for the rise of a new global center of science.

## 1. Introduction

The Nobel prizes in Physiology or Medicine, Physics, and Chemistry have attracted continued attention from the history of science and quantitative science and innovation perspectives. The former has predominantly focused on the social process shaping candidate selection, elucidating case by case the roles of the Nobel committees, Nobel Foundation, Royal Swedish Academy of Sciences, and Karolinska Institute [1–4]. The latter studies, in contrast, have examined the Nobel population with respect to more general attributes, including achievement age [5, 6], time lag between prize-winning work and awarding of the prize [7, 8], distribution of major science awards and collaboration networks in the years before and after each Nobel award [9–11], and distribution of laureate careers across universities and other research organizations [12]. These studies found that the mean age at which future laureates conducted their prize-winning research increased by about 10 years during the 20^th^ century, with prize-winning research before age 40 years becoming more and more rare [5]. They also found that the average waiting time has grown from 10 years in the 1920s to about 25 years in the 2000s. For this reason, these authors warned that “the prizewinners’ predicted average age for receiving the award is likely to exceed his or her projected life expectancy” [8]. Also, future laureates accumulate many other science awards before receiving the Nobel Prize, with theoreticians accruing more awards than experimentalists [9, 10]. Nobel laureates also had more collaboration partners before than after the award [11] and were “more equally distributed among the institutions where the prize-winning work was done and the Nobel Prize awarded than among the institutions where the Ph.D./M.D. was obtained” [12].

Several recent studies have addressed the bibliometric characterization of Nobel laureates. These investigations have revealed spillover effects for recipients in that “groundbreaking discoveries of Nobel Prize Laureates (…) also boost the citation rates of their previous publications” [13] and that the “average citation frequencies of landmark papers on new theories are far higher than those for an experimental method or for an invention” [14]. In cross-national comparisons, bibliometric indicators, such as the “numbers of publications, citations, and top 1 percent most-cited publications, correlate with the number of Nobel Prize achievements in several advanced countries,” leading to the conclusion that “Nobel Prize achievements can be used to validate bibliometric indicators” [15].

This paper contributes to a debated yet understudied topic: North America’s global leadership in science. First, there is some disagreement about when North America, and especially the United States, became the global center of science during the 20^th^ century. Some argue that research-intensive North American universities became globally competitive in the 1920s [16–18]. Others locate the shift after the Second World War, however, pointing to the considerable emigration of eminent European scientists after 1933 and the unprecedented growth of government-sponsored “Big Science” [19, 20]. Second, the enormous growth of scientific research in Asia since the 1990s, especially in China, has sparked discussions about the impending decline of North American hegemony in science [21–23].

This paper studies the changing global distribution of Nobel science laureates (n = 599) over 117 years, distinguishing three world regions: Asia-Pacific, Europe, and North America. We identify when global leadership in science shifted from Europe to North America and when the latter started to lose some of its global share, an indication of growing global competition at the beginning of the 21^st^ century. In this context, we examine institutional factors associated with North America’s scientific strength and more generally with dynamic science systems at the scale of world regions. Finally, we put our own results in perspective, using recent bibliometric evidence from comparative cross-national studies [24–27].

## 2. Material and methods

### 2.1. Materials

We compiled a dataset for all Nobel laureates who received the Nobel Prize in Physiology or Medicine, Physics, or Chemistry from 1901 to 2017 (n = 599). The primary source for our data compilation was the Nobel Foundation’s website, www.nobelprize.org. We collected data on: (a) the year, organization, and country of both the first and the highest academic degrees (HDs), the latter typically being either a medical doctor (M.D.) or a doctor of Philosophy (Ph.D.); (b) the year, organization, and country of where each laureate’s prize-winning work (PWR) was performed; (c) the year, organization, and country where the laureate worked when awarded the Nobel Prize (designated from here as NP); (d) whether the laureate had a Nobel mentor while a graduate student, postdoctoral researcher, or junior collaborator or during extended research sabbaticals (S1 Table); and (e) year of birth.

Regarding (a), we constructed highest academic degree variables that contain M.D. or Ph.D. information (n = 563); for those without the M.D. or Ph.D. (or equivalent), we used information from bachelor’s and master’s level degrees (n = 36). Regarding (b), we built our variables using publicly available data from [5]; these data were double-checked and extended for 2009–2017. Regarding (d), our definition of master–apprentice relations (S1 Table) extends available approaches in the literature. For example, [20] does not include extended research sabbaticals, whereas [28] includes Ph.D.-related apprenticeship only.

In cases where the Nobel Foundation’s website data were insufficient, we consulted other sources, including (in alphabetical order): American Association of Immunologists, American Chemical Society, American Institute of Physics, American National Biography, Encyclopedia Britannica, Howard Hughes Medical Institute, Leopoldina, Munzinger, National Academy of Sciences, Notable Names Database, and Royal Society. In addition, other printed sources were used [16, 29–33]. Finally, we also accessed dozens of university websites (not documented here).

Several organizations that existed in the 19^th^ and the 20^th^ centuries are now either closed or merged with other entities. In these cases, we applied the following two rules: (a) for organizations that were closed, we used the last available name (e.g., the last available name for the former Kaiser Wilhelm Institute for Cell Physiology was Max Planck Institute for Cell Physiology, which closed in 1972); and (b) for organizations that were merged with other entities, we used the organization that still exists (e.g., Owens College, founded in 1846, was merged with Manchester University).

### 2.2. Methods

We delineated three world regions: (1) Europe, including all 28 current European member states plus Norway and Switzerland; (2) North America, including Canada and the United States; and (3) Asia-Pacific, including Australia, China, India, Japan, Indonesia, and New Zealand. No country other than these six from the Asia-Pacific region has contributed to the Nobel population. For these three world regions, we determined the absolute and relative frequencies of Nobel laureates for the three career events (HD, PWR, NP). Hence, the distributions in all three career events, including HD and PWR, are mapped onto time periods defined by NP. The three regions are defined by the university or research organization in which HD, PWR, or NP occurred. Three countries are not covered by our regional delineation: Argentina, Israel, and Russia. However, their combined contribution to the Nobel population is very small: HD (3.2%), PWR (3.2%), and NP (2.8%). We also measured migration between regions across career events.

Then, we measured the number of master–apprentice relations for Europe and North America, first using consecutive 10-year NP periods, and second based on consecutive 10-year apprenticeship periods. If master laureates had not been apprentices themselves, they were positioned in their own HD period (S1–S6 Figs). Underlined names are mentors from outside the respective region. Because Nobel research often cuts across disciplinary boundaries [34, 35], we included cross-disciplinary master–apprentice relations (S1–S6 Figs).

We also measured the number of universities and research organizations hosting laureates for the first time (“newcomers”) across the three career events (HD, PWR, NP) for consecutive 10-year periods. In contrast to [36], who examined all occupational changes among laureates who had finished their academic careers, we focused on inter-organizational mobility that involved the three targeted significant career events (HD, PWR, NP). Our mobility measures are the sums of laureate moves from HD to PWR and/or from PWR to NP, respectively. The number of laureates across universities and research organizations had a skewed distribution in which few entities with many laureates were followed by a long tail of institutions having only a few laureates (S7 Fig).

Furthermore, we conducted an ordinary least squares (OLS) regression analysis on the total number of Nobel Prizes as a dependent variable, measured during the HD, PWR, and NP periods. For pragmatic reasons, we chose 5-year time windows; thus, observations for 22 periods were available. Because our variables are count variables, we also tested the robustness of the OLS regressions by using negative binomial regression models [37]. We performed all analyses using the freeware software packages R and Python.

## 3. Empirical results

**Fig 1:**
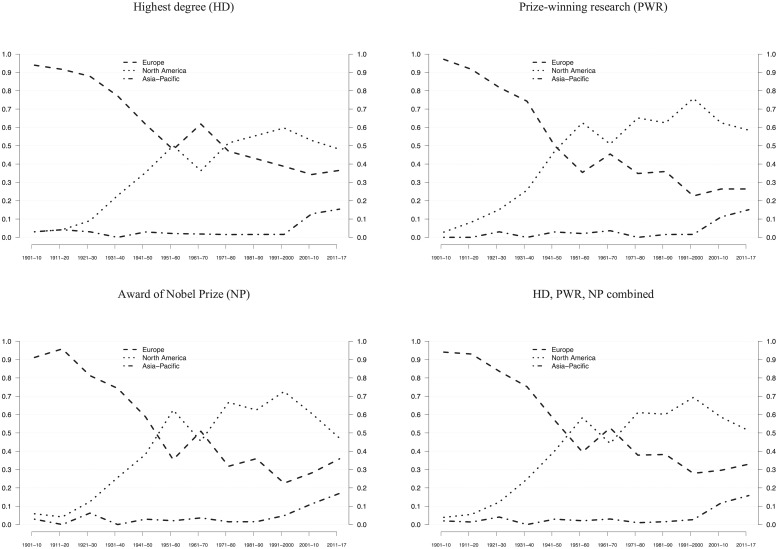
Global distribution of Nobel laureates. The relative frequencies of Nobel laureates across the three world regions, based on three career events (HD, PWR, NP). All observations refer to consecutive 10-year NP periods. Therefore, distributions in HD and PWR also are mapped onto NP periods. Lines are smoothened, using 3-period moving averages. The final period of 2000–2017 is weighted and thus comparable to earlier 10-year periods. Data from S2 Table are used.

We examined global scientific leadership in two ways. First, we measured the number of Nobel laureates across the three regions and across the three significant career events. Fig 1 is based on observations in consecutive 10-year periods, defined by NP. For example, observations in HD and PWR in 1931–1940 tell us where laureates who received the NP in that period had obtained their highest degree and conducted their prize-winning work.

We found four somewhat overlapping historical periods that characterize the shifting balance between the three world regions. In the first period, North America started to displace Europe as a stronghold of the Nobel population. This double decline/catch-up period lasted about 30 years (1920s to the 1940s) for PWR and the award of the NP, but about 40 years (1920s to 1950s) with regard to the education of future Nobel laureates (HD). The second period (1940s to 1960s) shows a transition, at the end of which North America had superseded Europe in all three career events (HD, PWR, NP). Similar to the first period, the transition to North American hegemony lasted about 30 years, ending in the 1960s for PWR and the award of the NP, whereas the transition took longer with regard to the education of future Nobel laureates (HD). Thus, the education of Nobel laureates remained a European stronghold longer than the particular work environments that ultimately led to the NP award.

The third period (1970s to 1990s) represents North American hegemony, when universities and other research organizations in North America educated up to 60 percent (HD) of all laureates and provided the work environment where roughly 75 percent of all laureates conducted their PWR and received the NP. North America’s shares started to decline in the 2000s, dropping to 48 percent (HD), 47 percent (PWR), and 58 percent (NP) in the 2010s. At the same time, Europe’s shares consolidated at 37 percent (HD), 36 percent (PWR), and 26 percent (NP), and Asia-Pacific’s shares grew considerably, reaching 15 percent (HD), 17 percent (PWR), and 15 percent (NP). However, contradicting speculations about China as a rising global science center, this growth resulted almost entirely from Japan. Hence, the fourth period (2000s and onwards) shows three developments: a relative North American decline, a moderate European rebound, and an Asia-Pacific catch-up. Compared to the North American catch-up (1920s-1950s), the more recent Asia-Pacific catch-up seems less pronounced. In this regard, one should consider the much stronger European hegemony in the early 20^th^ century compared to North America’s at the end of the 20^th^ century (Fig 1).

North America’s scientific strength is also reflected in patterns of inter-regional migration of scientists. Empirical studies have shown that North America not only imported academic talent during the transition period (1940s to 1960s) but also continued to do so during its hegemonic period (1970s to 1990s) [38] and later, in the post-hegemonic period (2000s and onwards) [39]. Hence, like its “trade deficit” in the world of commerce [40–42], North America has a “mobility deficit” in science, meaning that more foreign scientists are imported to (than exported from) North America [43]. The mobility to North America is typically motivated by improved opportunities for increased productivity and/or scientific impact [44, 45] and by career advancement [46].

**Fig 2:**
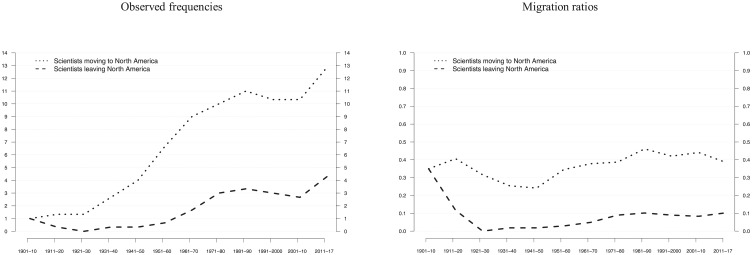
Nobel laureate migration to (and from) North America. Laureate migration in consecutive 10-year NP periods, using 3-period moving averages. Mobility includes moves from HD to PWR and/or from PWR to NP. Migration to (and from) North America includes scientists from Europe and Asia-Pacific in observed frequencies and ratios of observed to expected frequencies. Values for ratios <1 indicate that observed frequencies are smaller than expected frequencies. The final period of 2000–2017 is weighted and thus comparable to earlier 10-year periods. Data from S3 Table are used.

Based on these findings, we examined first the extent to which (future) laureates from Europe and the Asia-Pacific region moved to North America over time and vice versa. Fig 2 shows both the observed frequencies and the ratios of observed to expected frequencies. We found that during its hegemonic period (1970s to 1990s), North America attracted on average about 11 laureates every 10 years from Europe and the Asia-Pacific region, which is more than during the transition period (about 7 laureates). However, during the hegemonic period, there already was an increase in the absolute numbers of laureates leaving North America for Europe or Asia-Pacific. The net migration effect for North America remained approximately constant over the hegemonic and the post-hegemonic periods, when North America still attracted around 12 laureates per decade (S3 Table). The comparison with expected frequencies, based on random sampling, supports this interpretation of a continued attractiveness of the North American science system (Fig 2).

Second, we identified all (future) Nobel laureates who had established master–apprentice relations with other laureates, typically before the laureate masters were awarded the NP. The apprentice laureates either studied as graduate students or worked with the masters as postdoctoral researchers or junior collaborators or during extended research sabbaticals (S1 Table). These relations are relevant because they indicate effective transfer of the ability to conduct ground-breaking research from one generation of scientists to another. Such capabilities are an important element of global leadership in science ([20] pp. 96–143).

**Fig 3:**
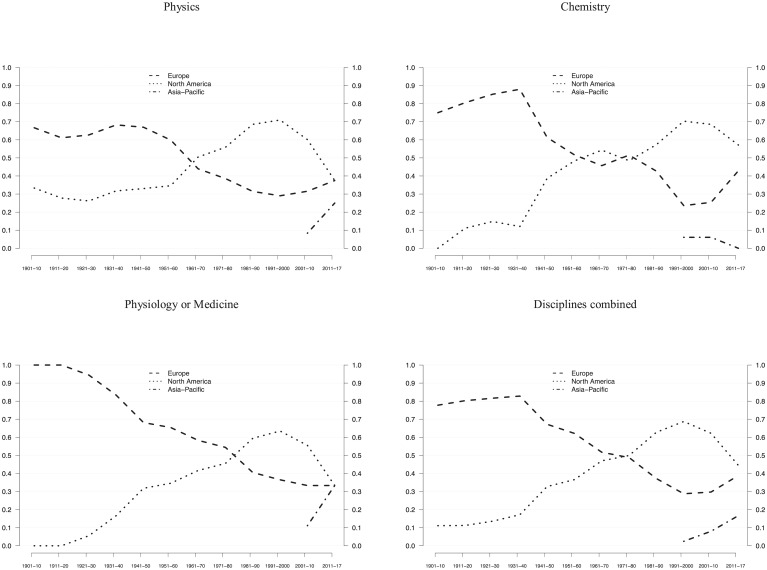
Master–apprentice relations among Nobel laureates. The relative frequencies of master–apprentice relations for the three science disciplines each and combined, in consecutive 10-year NP periods, using 3-period moving averages. The final period of 2000–2017 is weighted and thus comparable to earlier 10-year periods. Data from S4 Table are used.

If all three disciplines are considered together, Europe held the majority of master–apprentice relations until the 1970s (Fig 3). Yet, in accordance with the findings displayed in Fig 1, Europe’s share already had declined in the 1930s, during the catch-up period in which North America started to displace Europe as leading scientific region. A more fine-grained view shows that during the second historical period (transition to North American hegemony, 1940s to 1960s), transatlantic master–apprentice pairs were particularly frequent (S1–S6 Figs). During its hegemony (1970s to 1990s), North America continued to accumulate laureate apprentices, reaching its highest level in the 1990s (68%). Then, in the fourth period, North America’s share of apprentice—master relations plummeted, reaching 42 percent in the 2010s. At the same time, Europe consolidated (37%) and the Asia-Pacific region increased (16%) their respective shares. With regard to the most recent period, the absolute decline of master–apprenticeship relations (S4 Table) should be interpreted with caution because data on future laureates are not complete given the time lag between PWR and NP (S1–S6 Figs).

Taken together, these descriptive results suggest that North America’s rise as global power in science started in the 1920s and that its leadership position was consolidated in the 1970s. North America’s hegemony in science lasted about 30 years (1970s to 1990s), preceded by a transition period in which it superseded Europe (1940s to 1960s). Yet, beginning in the 2000s, we identified a period of increasing global competition. First, North America’s global share of laureates dropped significantly, across both the three major career events (HD, PWR, NP) and the three disciplines (Physics, Chemistry, and Physiology or Medicine). Second and related, the continuity among consecutive generations of prize-winning scientists in North America seems to have weakened. In the 2010s –and thus for the first time since the 1960s –Europe and the Asia-Pacific region together might have more laureate apprentices than North America, but this observation depends on the award of future NPs.

Our descriptive findings are further elaborated by a regression analysis on North America (S5 Table). The result shows that the number of master–apprentice relations explains more than 80 percent of the variation in the number of NPs across the three career states (HD+PWR+NP). Most interestingly, the relationship between both variables follows an inverted U-shape, as shown by the negative coefficient of the squared term. Thus, although an increase in master–apprentice relations increases the number of NPs across the three career events in a given time (here: 5-year periods) to a certain threshold (here: about 22), it starts to decrease for all n>22 (Model 2).

We also controlled for the transition period (1940s to 1960s) and/or the hegemonic period (1970s to 1990s), but even though the directions of these variables were plausible, none of them was statistically significant (Models 3, 4). Furthermore, we tested the robustness of our models by including a modified NP variable (PWR+NP), and by using negative binomial instead of OLS models. The results remained stable (and are available upon request to the first author). Finally, conducting the same analysis for Europe did not yield meaningful results.

North America’s strength in science has often been attributed to the organizational design of its research universities, most notably the combination of collegial academic departments, research-oriented graduate schools, and strategic leadership of university presidents [17, 47, 48]. In addition, comparative analyses have shown that North American universities compete more actively for scientific talent [17, 18] and thus adopt scientific innovations faster than European institutions [49, 50]. Taking up the latter argument, we probed whether the level of competition among universities and research organizations has changed over time. As a measure, we examined the share of “newcomers” entering the inter-organizational competition for scientific talent: research organizations hosting laureates for the first time in either of the three career events (HD, PWR, NP). The share of newcomer organizations indicates the degree of organizational renewal (versus organizational inertia) within the Nobel population.

**Fig 4:**
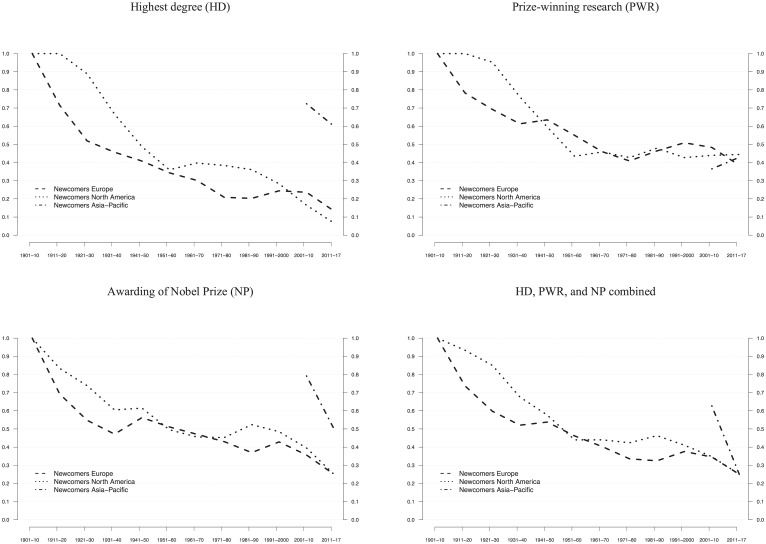
Newcomer organizations across world regions. The relative frequency of organizations hosting Nobel laureates for the first time (“newcomers”) across the three career events (HD, PWR, NP) in consecutive 10-year NP periods, using 3-period moving averages. Lines for Asia-Pacific start in 2001, when the total number of organizations was >5. The final period of 2000–2017 is weighted and thus comparable to earlier 10-year periods. Data from S6 Table are used.

Fig 4 shows that, if all three career stages combined are considered, there is a general trend of decreasing shares of newcomer organizations across the three regions over the entire observation period (1901–2017). Two findings are noteworthy in this regard. First, the share of newcomer organizations across all career events combined is significantly higher in North America compared to Europe not only during the catch-up period (1920s to 1940s) but also during the hegemonic period (1970s to 1990s). This finding might point to differential patterns in the expansion of higher education between both regions (Fig 4). Second, the decrease in newcomer organizations is less pronounced for PWR compared to HD and NP. All three regions converge towards a relatively high level of newcomer organizations for PWR towards the end of the observation period (40%-50%, Fig 4), and this organizational dynamic has persisted in both North America and Europe during the hegemonic and post-hegemonic periods. We conclude from this finding that regarding the organizational context in which prize-winning research is conducted, the three world regions are clearly competitors for scientific leadership.

## 4. Discussion

Our results based on the Nobel population can be summarized in three points. First, North America’s rise as a global power in science started in the 1920s. Following a transition period (1940s to 1960s), its leadership position began in the 1970s and lasted about 30 years (1970s to 1990s). Yet, in the 2000s, North America’s hegemony started to be challenged again by more inter-regional competition: its global share of laureates dropped, across the three major career events (HD, PWR, NP) and across the three disciplines (Physics, Chemistry, and Physiology or Medicine). While inter-regional migration of future laureates is no longer a one-way road, North America has remained the most attractive destination. Second, although North America was effective in transferring capacities for conducting ground-breaking research from one generation of scientists to another for a long time (via master–apprentice relations), Europe and the Asia-Pacific region seem to be catching up. Third, North America’s scientific hegemony has often been attributed to the organizational design of its research universities, which compete more actively for scientific talent and adopt scientific innovations faster than universities and research institutions in Europe (and elsewhere). Taking newcomer organizations as an indicator for organizational renewal, we found that both Europe and North America are more dynamic in the organizational context in which prize-winning research is conducted. Newcomers are less important among the set of universities and research organizations where future laureates either receive their Ph.D. and/or their NP.

Our results on the Nobel population are broadly corroborated by recent bibliometric comparisons that show a declining citation impact gap between North America and other world regions since the 2000s [27, 51, 52]. In particular, several cross-country bibliometric comparisons show that while North America was in the lead until the early 2000s [26], its share among both the 1 percent and the 10 percent most highly cited publications has declined since the mid-2000s. In the meantime, countries from Europe and the Asia-Pacific region have increased their shares, respectively [24, 25, 27]. Therefore, our analysis complements existing bibliometric cross-country and cross-regional comparisons.

Future research should focus on the social process of how Nobel laureates are chosen from a quantitative perspective, including the roles of the Nobel committees, Nobel Foundation, Royal Swedish Academy of Sciences, and Karolinska Institute, and of research institutions and nominators outside Scandinavia. In that regard, it is important to understand that the Nobel Archives are closed for 50 years after the award. Therefore, future research should consider the so-called “Nomination Database” that the Nobel Foundation recently made available. This archive contains all nominees and nominators in Chemistry, Physics, and Physiology or Medicine for the years 1901–1966.

Another line of future inquiry could focus on systematic cross-regional comparisons based on archival data on R&D expenditure and the scientific work force at the national and/or regional level. These data should include measures such as number of inhabitants, number of employed scientists, gross domestic product, or patent applications and citations. The current analysis, like earlier comparisons, builds on both absolute and relative measures of scientific performance without reference to the national and/or regional investments in science and technology. It would be interesting to know whether and to what extent the overall picture, as shown here (and in other bibliometric comparisons), would change if such measures were to be taken into account.

## Supporting information

S1 Fig Master–apprentice relations in Physics (Europe). Master–apprentice relations among Nobel laureates in Physics (Europe). Underlined names are from other regions.
(EPS)

S2 Fig Master–apprentice relations in Physics (North America). Master–apprentice relations among Nobel laureates Physics (North America). Underlined names are from other regions.
(EPS)

S3 Fig Master–apprentice relations in Chemistry (Europe). Master–apprentice relations among Nobel laureates in Chemistry (Europe). Underlined names are from other regions.
(EPS)

S4 Fig Master–apprentice relations in Chemistry (North America). Master–apprentice relations among Nobel laureates in Chemistry (North America). Underlined names are from other regions.
(EPS)

S5 Fig Master–apprentice relations in Physiology or Medicine (Europe). Master–apprentice relations among Nobel laureates in Physiology or Medicine (Europe). Underlined names are from other regions.
(EPS)

S6 Fig Master–apprentice relations in Physiology or Medicine (North America). Master–apprentice relations among Nobel laureates in Physiology or Medicine (North America). Underlined names are from other regions.
(EPS)

S7 Fig Distribution of Nobel laureates across universities and research organizations. The distribution of Nobel laureates across universities and research organizations for the three career events (HD, PWR, NP), 1901–2017. Y-axis shows the number of Nobel laureates, and x-axis shows the number of universities and research organizations with at least one entry in either HD, PWR, or NP. For better graphical layout (shorter x-axis), three outlier values are not displayed: 67 organizations each have 1 HD entry, 132 organizations each have 1 NWR entry, and 128 organizations each have 1 NP entry.
(EPS)

S1 Table Types of master–apprentice relations. Four categories of master–apprentice relations and relevant examples from the Nobel Foundation’s website.
(DOCX)

S2 Table Nobel laureates across world regions. Absolute frequencies of Nobel laureates across the three world regions. The final period of 2000–2017 is weighted and thus comparable to earlier 10-year periods. Relative frequencies (moving averages) are shown in Fig 1.
(DOCX)

S3 Table Nobel laureate mobility to (and from) North America. Observed and expected frequencies of laureate mobility. Mobility includes moves from HD to PWR and/or from PWR to NP. Mobility to (and from) North America includes scientists from Europe and Asia-Pacific. Expected frequencies are based on random sampling. The final period of 2000–2017 is weighted and thus comparable to earlier 10-year periods. Both observed frequencies and ratios between observed/expected frequencies are shown in Fig 2.
(DOCX)

S4 Table Master–apprentice relations across disciplines. Absolute frequencies of master–apprentice relations across the three disciplines. The final period of 2000–2017 is weighted and thus comparable to earlier 10-year periods. Relative frequencies (moving averages) are shown in Fig 3.
(DOCX)

S5 Table OLS regression results (North America). OLS regression results (pooled) for 5-year time windows beginning in 1901. Thus, the models cover 22 time periods. Parentheses show standard errors. Data from S2 and S4 Tables are used.
(DOCX)

S6 Table Newcomer organizations across world regions. Absolute frequencies of organizations hosting Nobel laureates for the first time (“newcomers”). The final period of 2000–2017 is weighted and thus comparable to earlier 10-year periods. Relative frequencies (moving averages) are shown in Fig 4.
(DOCX)
