# Correction: From North American hegemony to global competition for scientific leadership? Insights from the Nobel population

**DOI:** 10.1371/journal.pone.0219582

**Published:** 2019-07-09

**Authors:** Thomas Heinze, Arlette Jappe, David Pithan

The graph for S4 Fig is incorrect and the legends for S4–S6 Figs are incorrect. Please see the corrected S4–S6 Figs below.

## Supporting information

S4 Fig Master–apprentice relations in Chemistry (North America). Master–apprentice relations among Nobel laureates in Chemistry (North America). Underlined names are from other regions.
(EPS)

S5 Fig Master–apprentice relations in Physiology or Medicine (Europe). Master–apprentice relations among Nobel laureates in Physiology or Medicine (Europe). Underlined names are from other regions.
(EPS)

S6 Fig Master–apprentice relations in Physiology or Medicine (North America). Master–apprentice relations among Nobel laureates in Physiology or Medicine (North America). Underlined names are from other regions.
(EPS)
