# Supplementary material for: From North American hegemony to global competition for scientific leadership? Insights from the Nobel population
Source: PLoS One. 2019 Apr 3;14(4):e0213916. doi: 10.1371/journal.pone.0213916 (PMC6447154; doi:10.1371/journal.pone.0213916)
Supplement: S1 Table — Four categories of master–apprentice relations and relevant examples from the Nobel Foundation’s website. (DOCX) [file pone.0213916.s008.docx]

**S1 Table. Types of master–apprentice relations**

| **Graduate Student** | **Postdoctoral Fellow** | **Junior Collaborator** | **Extended Research Sabbatical** |
| --- | --- | --- | --- |
| Otto Warburg (mentee), Emil Fischer (mentor): “Otto studied chemistry under the great Emil Fischer, and gained the degree, Doctor of Chemistry (…). Warburg’s early researches with Fischer were in the polypeptide field.”  <http://www.nobelprize.org/nobel_prizes/medicine/laureates/1931/warburg-bio.html> | Serge Haroche (mentee), Arthur Schalow (mentor): “I chose to become a postdoctoral fellow at Stanford University, in the laboratory of Arthur Schawlow.”  <https://www.nobelprize.org/nobel_prizes/physics/laureates/2012/haroche-bio.html> | John Eccles (mentee), Charles Sherrington (mentor): “Later from 1928 to 1931 he was research assistant to Sherrington, there being eight papers published conjointly.”  <http://www.nobelprize.org/nobel_prizes/medicine/laureates/1963/eccles-bio.html> | Paul Boyer (mentee), Hugo Thorell (mentor): “A sabbatical period on a Guggen­heim Fellowship (…) was especially rewarding. (…) I did research at (...)the Nobel Medical Institute, working with Hugo Theorell’s group.”  <https://www.nobelprize.org/nobel_prizes/chemistry/laureates/1997/boyer-bio.html> |
| Kurt Alder (mentee), Otto Diels (mentor): “he obtained his degree of Ph.D. in 1926. The thesis for the doctorate, on which Alder worked under O. Diels, was entitled: Über die Ursachen der Azoesterreaktion.”  <https://www.nobelprize.org/nobel_prizes/chemistry/laureates/1950/alder-bio.html> | Gerhard Herzberg (mentee), Max Born/James Franck (mentors): “From 1928 to 1930 he carried out post-doctorate work at the University of Göttingen under James Franck and Max Born and the University of Bristol.”  <https://www.nobelprize.org/nobel_prizes/chemistry/laureates/1971/herzberg-bio.html> | Ei-ichi Negishi (mentee), Herbert Brown (mentor): “which eventually led me to join H.C. Brown’s group (…) as his assistant with the rank of instructor for four more years (1968–1972).”  <https://www.nobelprize.org/nobel_prizes/chemistry/laureates/2010/negishi-bio.html> | Elias Corey (mentee), Karl Berg­ström (mentor): “I received a Guggenheim fellowship (...) It was at Lund, in Bergström’s Department, that I became intrigued by the prostaglandins.”  <https://www.nobelprize.org/nobel_prizes/chemistry/laureates/1990/corey-bio.html> |

Four categories of master–apprentice relations and relevant examples from the Nobel Foundation’s website.
