# Supplementary material for: From North American hegemony to global competition for scientific leadership? Insights from the Nobel population
Source: PLoS One. 2019 Apr 3;14(4):e0213916. doi: 10.1371/journal.pone.0213916 (PMC6447154; doi:10.1371/journal.pone.0213916)
Supplement: S2 Table — Absolute frequencies of Nobel laureates across the three world regions. The final period of 2000–2017 is weighted and thus comparable to earlier 10-year periods. Relative frequencies (moving averages) are shown in Fig 1. (DOCX) [file pone.0213916.s009.docx]

S2 Table. Nobel laureates across world regions

| Award Period | Europe | North America | Asia-Pacific |
| --- | --- | --- | --- |
|  | Highest degree (HD) | | |
| 1901-1910 | 31 | 1 | 1 |
| 1911-1920 | 22 | 1 | 1 |
| 1921-1930 | 29 | 3 | 1 |
| 1931-1940 | 27 | 8 | 0 |
| 1941-1950 | 21 | 12 | 1 |
| 1951-1960 | 23 | 24 | 1 |
| 1961-1970 | 34 | 20 | 1 |
| 1971-1980 | 31 | 34 | 1 |
| 1981-1990 | 27 | 35 | 1 |
| 1991-2000 | 24 | 37 | 1 |
| 2001-2010 | 24 | 37 | 9 |
| 2011-2017 | 27 | 36 | 11 |
|  | Prize-winning research (PWR) | | |
| 1901-1910 | 31 | 2 | 1 |
| 1911-1920 | 23 | 1 | 0 |
| 1921-1930 | 26 | 4 | 2 |
| 1931-1940 | 26 | 9 | 0 |
| 1941-1950 | 20 | 13 | 1 |
| 1951-1960 | 17 | 30 | 1 |
| 1961-1970 | 28 | 25 | 2 |
| 1971-1980 | 21 | 44 | 1 |
| 1981-1990 | 23 | 40 | 1 |
| 1991-2000 | 14 | 45 | 3 |
| 2001-2010 | 20 | 43 | 8 |
| 2011-2017 | 27 | 36 | 13 |
|  | Nobel Prize (NP) | | |
| 1901-1910 | 34 | 1 | 0 |
| 1911-1920 | 22 | 2 | 0 |
| 1921-1930 | 27 | 5 | 1 |
| 1931-1940 | 26 | 9 | 0 |
| 1941-1950 | 17 | 16 | 1 |
| 1951-1960 | 17 | 30 | 1 |
| 1961-1970 | 25 | 28 | 2 |
| 1971-1980 | 23 | 43 | 0 |
| 1981-1990 | 23 | 40 | 1 |
| 1991-2000 | 14 | 47 | 1 |
| 2001-2010 | 19 | 45 | 8 |
| 2011-2017 | 20 | 44 | 11 |

Absolute frequencies of Nobel laureates across the three world regions. The final period of 2000–2017 is weighted and thus comparable to earlier 10-year periods. Relative frequencies (moving averages) are shown in Fig 1.
