# Supplementary material for: From North American hegemony to global competition for scientific leadership? Insights from the Nobel population
Source: PLoS One. 2019 Apr 3;14(4):e0213916. doi: 10.1371/journal.pone.0213916 (PMC6447154; doi:10.1371/journal.pone.0213916)
Supplement: S3 Table — Observed and expected frequencies of laureate mobility. Mobility includes moves from HD to PWR and/or from PWR to NP. Mobility to (and from) North America includes scientists from Europe and Asia-Pacific. Expected frequencies are based on random sampling. The final period of 2000–2017 is weighted and thus comparable to earlier 10-year periods. Both observed frequencies and ratios between observed/expected frequencies are shown in Fig 2. (DOCX) [file pone.0213916.s010.docx]

S3 Table. Nobel laureate mobility to (and from) North America

|  | Mobility to North America | Mobility from North America |
| --- | --- | --- |
|  | Observed Frequencies | Observed Frequencies |
| 1901-1910 | 1 | 1 |
| 1911-1920 | 1 | 0 |
| 1921-1930 | 2 | 0 |
| 1931-1940 | 1 | 0 |
| 1941-1950 | 5 | 1 |
| 1951-1960 | 6 | 0 |
| 1961-1970 | 9 | 1 |
| 1971-1980 | 12 | 4 |
| 1981-1990 | 9 | 4 |
| 1991-2000 | 12 | 2 |
| 2001-2010 | 10 | 3 |
| 2011-2017 | 13 | 4 |
|  | Expected Frequencies | Expected Frequencies |
| 1901-1910 | 2,9 | 2,9 |
| 1911-1920 | 1,9 | 2,9 |
| 1921-1930 | 6,0 | 8,0 |
| 1931-1940 | 12,6 | 13,6 |
| 1941-1950 | 14,3 | 18,3 |
| 1951-1960 | 20,3 | 26,3 |
| 1961-1970 | 23,1 | 31,1 |
| 1971-1980 | 26,8 | 34,8 |
| 1981-1990 | 27,8 | 32,8 |
| 1991-2000 | 19,7 | 29,7 |
| 2001-2010 | 30,6 | 37,6 |
| 2011-2017 | 33,3 | 41,9 |

Observed and expected frequencies of laureate mobility. Mobility includes moves from HD to PWR and/or from PWR to NP. Mobility to (and from) North America includes scientists from Europe and Asia-Pacific. Expected frequencies are based on random sampling. The final period of 2000–2017 is weighted and thus comparable to earlier 10-year periods. Both observed frequencies and ratios between observed/expected frequencies are shown in Fig 2.
