# Supplementary material for: From North American hegemony to global competition for scientific leadership? Insights from the Nobel population
Source: PLoS One. 2019 Apr 3;14(4):e0213916. doi: 10.1371/journal.pone.0213916 (PMC6447154; doi:10.1371/journal.pone.0213916)
Supplement: S4 Table — Absolute frequencies of master–apprentice relations across the three disciplines. The final period of 2000–2017 is weighted and thus comparable to earlier 10-year periods. Relative frequencies (moving averages) are shown in Fig 3. (DOCX) [file pone.0213916.s011.docx]

S4 Table. Master–apprentice relations across disciplines

| Award Period | Europe | North America | Asia-Pacific |
| --- | --- | --- | --- |
|  | Physics | | |
| 1901-1910 | 2 | 1 | 0 |
| 1911-1920 | 2 | 0 | 1 |
| 1921-1930 | 1 | 1 | 0 |
| 1931-1940 | 5 | 2 | 0 |
| 1941-1950 | 5 | 1 | 0 |
| 1951-1960 | 6 | 7 | 0 |
| 1961-1970 | 3 | 2 | 1 |
| 1971-1980 | 5 | 9 | 0 |
| 1981-1990 | 3 | 7 | 0 |
| 1991-2000 | 2 | 5 | 0 |
| 2001-2010 | 2 | 5 | 0 |
| 2011-2017 | 4 | 4 | 3 |
|  | Chemistry | | |
| 1901-1910 | 3 | 0 | 1 |
| 1911-1920 | 2 | 1 | 0 |
| 1921-1930 | 6 | 0 | 0 |
| 1931-1940 | 8 | 1 | 0 |
| 1941-1950 | 6 | 2 | 0 |
| 1951-1960 | 1 | 4 | 0 |
| 1961-1970 | 3 | 2 | 0 |
| 1971-1980 | 4 | 3 | 0 |
| 1981-1990 | 3 | 5 | 0 |
| 1991-2000 | 2 | 4 | 0 |
| 2001-2010 | 0 | 9 | 2 |
| 2011-2017 | 4 | 6 | 0 |
|  | Physiology or Medicine | | |
| 1901-1910 | 2 | 0 | 0 |
| 1911-1920 | 1 | 0 | 0 |
| 1921-1930 | 4 | 0 | 0 |
| 1931-1940 | 5 | 1 | 0 |
| 1941-1950 | 4 | 2 | 0 |
| 1951-1960 | 6 | 5 | 0 |
| 1961-1970 | 12 | 4 | 0 |
| 1971-1980 | 5 | 6 | 0 |
| 1981-1990 | 3 | 4 | 0 |
| 1991-2000 | 3 | 6 | 0 |
| 2001-2010 | 3 | 6 | 0 |
| 2011-2017 | 1 | 1 | 1 |

Absolute frequencies of master–apprentice relations across the three disciplines. The final period of 2000–2017 is weighted and thus comparable to earlier 10-year periods. Relative frequencies (moving averages) are shown in Fig 3.
