# Supplementary material for: From North American hegemony to global competition for scientific leadership? Insights from the Nobel population
Source: PLoS One. 2019 Apr 3;14(4):e0213916. doi: 10.1371/journal.pone.0213916 (PMC6447154; doi:10.1371/journal.pone.0213916)
Supplement: S5 Table — OLS regression results (pooled) for 5-year time windows beginning in 1901. Thus, the models cover 22 time periods. Parentheses show standard errors. Data from S2 and S4 Tables are used. (DOCX) [file pone.0213916.s012.docx]

S5 Table. OLS regression results (North America)

Dependent Variable: Number of Nobel Prizes (5-year period), measured for all three career events (HD+PWR+HD).

Independent Variables: Number of apprentice-master relations (5-year period), AM-Relations squared (5-year period), Transition Period (1941-1960), Hegemonic Period (1961-1999).

Model 1 Model 2 Model 3 Model 4

(Intercept) 4.4217 -2.9365 -2.5934 -2.3659

(3.9762) (4.5944) (4.3907) (4.5096)

AM-Relations 6.3124*** 12.2486*** 13.8666*** 13.2624***

(0.6388) (2.4220) (2.4970) (2.8553)

AM-Relations^2^ -0.5629* -0.7175** -0.6769*

(0.2232) (0.2314) (0.2515)

Transition Period -8.8263 -7.4121

(5.1434) (6.0526)

Hegemony Period 3.0653

(6.5199)

Adjusted R-squared 0.8207 0.8523 0.8654 0.8596

Significance codes: 0.001 ‘***’ 0.01 ‘**’ 0.05 ‘*’

N=22 observations

OLS regression results (pooled) for 5-year time windows beginning in 1901. Thus, the models cover 22 time periods. Parentheses show standard errors. Data from S2 Table and S4 Table are used.
