# Supplementary material for: From North American hegemony to global competition for scientific leadership? Insights from the Nobel population
Source: PLoS One. 2019 Apr 3;14(4):e0213916. doi: 10.1371/journal.pone.0213916 (PMC6447154; doi:10.1371/journal.pone.0213916)
Supplement: S6 Table — Absolute frequencies of organizations hosting Nobel laureates for the first time (“newcomers”). The final period of 2000–2017 is weighted and thus comparable to earlier 10-year periods. Relative frequencies (moving averages) are shown in Fig 4. (DOCX) [file pone.0213916.s013.docx]

S6 Table. Newcomer organizations across world regions

| Award Period | Europe | North America | Asia-Pacific |
| --- | --- | --- | --- |
|  | Highest degree (HD) | | |
| 1901-1910 | 23 | 1 | 1 |
| 1911-1920 | 9 | 1 | 1 |
| 1921-1930 | 14 | 3 | 1 |
| 1931-1940 | 7 | 4 | 0 |
| 1941-1950 | 6 | 2 | 1 |
| 1951-1960 | 7 | 6 | 1 |
| 1961-1970 | 5 | 4 | 1 |
| 1971-1980 | 5 | 8 | 0 |
| 1981-1990 | 3 | 8 | 1 |
| 1991-2000 | 4 | 5 | 1 |
| 2001-2010 | 6 | 4 | 4 |
| 2011-2017 | 3 | 1 | 4 |
|  | Prize-winning research (PWR) | | |
| 1901-1910 | 24 | 2 | 1 |
| 1911-1920 | 16 | 1 | 0 |
| 1921-1930 | 11 | 3 | 2 |
| 1931-1940 | 16 | 6 | 0 |
| 1941-1950 | 9 | 3 | 1 |
| 1951-1960 | 8 | 8 | 1 |
| 1961-1970 | 8 | 6 | 2 |
| 1971-1980 | 5 | 15 | 1 |
| 1981-1990 | 6 | 11 | 0 |
| 1991-2000 | 7 | 18 | 0 |
| 2001-2010 | 6 | 10 | 4 |
| 2011-2017 | 7 | 11 | 4 |
|  | Nobel Prize (NP) | | |
| 1901-1910 | 28 | 1 | 0 |
| 1911-1920 | 16 | 2 | 0 |
| 1921-1930 | 6 | 2 | 1 |
| 1931-1940 | 13 | 5 | 0 |
| 1941-1950 | 8 | 6 | 1 |
| 1951-1960 | 10 | 9 | 1 |
| 1961-1970 | 7 | 5 | 2 |
| 1971-1980 | 7 | 12 | 0 |
| 1981-1990 | 7 | 13 | 0 |
| 1991-2000 | 3 | 16 | 1 |
| 2001-2010 | 10 | 12 | 7 |
| 2011-2017 | 4 | 9 | 6 |

Absolute frequencies of organizations hosting Nobel laureates for the first time (“newcomers”). The final period of 2000–2017 is weighted and thus comparable to earlier 10-year periods. Relative frequencies (moving averages) are shown in Fig 4.
